# Protective Effects of Extracts from *Fructus rhodomyrti* against Oxidative DNA Damage *In Vitro* and *In Vivo*

**DOI:** 10.1155/2013/507407

**Published:** 2013-09-05

**Authors:** Yuebin Ke, Xinyun Xu, Shuang Wu, Juan Huang, Yijie Geng, Hara Misra, Yunbo Li

**Affiliations:** ^1^Key Laboratory of Molecular Biology of Shenzhen, Shenzhen Center for Disease Control and Prevention, Shenzhen 518055, China; ^2^Department of Biomedical Sciences and Pathobiology, Virginia Polytechnic Institute and State University, Blacksburg, VA 26060, USA

## Abstract

*Objective*. To evaluate the potential protective effects of extracts from *Fructus rhodomyrti* (FR) against oxidative DNA damage using a cellular system and the antioxidant ability on *potassium* bromate- (KBrO_3_-) mediated oxidative stress in rats. *Methods*. The effects of FR on DNA damage induced by hydrogen peroxide (H_2_O_2_) were evaluated by comet assay in primary spleen lymphocytes cultures. The effects of FR on the activities of SOD, CAT, and GPx and the levels of GSH, hydroperoxides, and 8-OHdG were determined in the plasma and tissues of rats treated with KBrO_3_. *Results*. FR was shown to effectively protect against DNA damage induced by H_2_O_2_ *in vitro*, and the maximum protective effect was observed when FR was diluted 20 times. Endogenous antioxidant status, namely, the activities of SOD, CAT, and GPx and the levels of GSH were significantly decreased in the plasma, the liver, and the kidney of the KBrO_3_-treated rats, while the pretreatment of FR prevented the decreases of these parameters. In addition, the pretreatment of FR was also able to prevent KBrO_3_-induced increases in the levels of hydroperoxides and 8-OHdG in the plasma, the liver, and the kidney in rats. *Conclusions*. Our findings suggested that FR might act as a chemopreventive agent with antioxidant properties offering effective protection against oxidative DNA damage in a concentration-dependent manner *in vitro* and *in vivo*.

## 1. Introduction

Plants have played significant roles in maintaining human health and improving the quality of human life for thousands of years [1, 2]*. Fructus rhodomyrti* (FR) is the fruit of the *Rhodomyrtus tomentosa* growing on knolls in wilderness and widely distributed in Guangdong, Guangxi, Yunnan, Fujian, and Taiwan. FR has been used for the production of drinks and wine. FR is a traditional Chinese medicine material with antihepatitis property [3]. 

Cells that live in an oxygen-rich environment are inundated with various endogenous and exogenous sources of reactive oxygen species (ROS) [4]. The most important target for ROS in the carcinogenesis process is DNA [5, 6]. Irreparable DNA damage is involved in carcinogenesis, aging, and other degenerative diseases [4, 7]. However, enzymatic and nonenzymatic systems, which preserve the oxidant/antioxidant status, are disrupted during oxidative stress, a metabolic derangement due to an imbalance caused by excessive generation of ROS or a diminished capacity of the antioxidant defense system. Dietary factors and natural antioxidants that reduce the impact of ROS can protect DNA damage and thus reduce the risk of cancers [8, 9].

Hydrogen peroxide (H_2_O_2_) and potassium bromate (KBrO_3_) are commonly used to induce oxidative damage [10–12]. KBrO_3_ is a widely used food additive, a water disinfection by-product, and a known nephrotoxic agent. Cellular proliferation was enhanced in the kidney due to oxidative stress generated by KBrO_3_. It has also been reported that KBrO_3_ increased the levels of 8-hydroxydeoxyguanosine (8-OHdG), an oxidative DNA adduct, suggesting that it can indirectly induce DNA modifications by oxygen radicals that are involved in carcinogenesis [9, 13].

In a previous study, FR has been confirmed abundant in flavonoid glycosides and phenols, and the flavonoids extracts of  FR displayed an antiradical action and antioxidant effects in serum of rats [3]. However, the biological effects of FR remained poorly understood so far. It is unclear whether or not FR has any protective effects against oxidative DNA damage and antioxidant effects in target organs induced by xenobiotics. In the present study, the potential protective effects of extracts from FR against oxidative DNA damage *in vitro* and oxidative stress induced by KBrO_3_ *in vivo* were explored. 

## 2. Materials and Methods

### 2.1. Materials and Equipments

Low melting point agarose, Triton-X100, and sodium lauroylsarcosine were purchased from Sigma Company (USA). The normal melting point agarose (NMPA), RPMI1640 medium, and Tris were purchased from Promega Company. Neonatal calf serum was purchased from Beijing Bangding Company, and H_2_O_2_ (analytically pure) was purchased from the National Institute for the Control of Pharmaceutical and Biological Products. The antibody against 8-OHdG and 8-OHdG enzyme-linked immunosorbent assay (ELISA) kits was purchased from JaICA (Japan). The confocal laser scanning microscope was purchased from Leica (Germany), and PAC200 transfer electrophoresis instrument was the product of BIO-RAD Company (USA). VE-186 transfer electrophoresis tank was purchased from Shanghai Jinpeng Analytical Instrument Company.

### 2.2. Preparation of FR Extract

FR originated in Shaoguan, Guangdong, was provided by Traditional Chinese Medicine University of Guangzhou. One kilogram of FR was ground and extracted with 2 L of distilled water at 4°C. After 24 h, the extracts were centrifuged at 15,000 ×g three times. The freeze dry sample was reextracted with 1 L of distilled water. The reextracted sample was extracted with 100% ethanol (1 : 4, *v*/*v*), and then the ethanol-insoluble fraction was collected and freeze-dried. The dried fraction was extracted with distilled water for further experiments. Stock solution (1 g/mL) was prepared with phosphate buffer solution (PBS) before use, boiled for 30 min, and then kept at 4°C. Appropriate concentrations were adjusted before use. The experimental concentration was 1 g/mL. Saline (0.85% NaCl) was used as a control.

### 2.3. Animals

Male albino rats (130–150 g) of Wistar strain were obtained from the Medical Experimental Animal Center of Sun Yat-Sen University, Guangzhou, China. The rats were housed in polypropylene cages in groups of six rats per cage in a room maintaining of 25 ± 2°C and a relative humidity of 40–70% with a 12 h light/dark cycle. The rats were allowed acclimatizing for one week before the experiments and had free access to standard laboratory feed and water ad libitum. The rats were sacrificed according to the guidelines of the current laws of Ethical Committee for the purpose of control and supervision of experiments on animals in China.

### 2.4. Culture of Spleen Lymphocytes

Sterile isolation of the intact spleen was immersed in PBS solution at 37°C. The spleen capsule and fat composition were removed. One mm^3^ of spleen tissue was cut off, digested in pancreatin (1-2 min), and then placed in the metal filter net with PBS (pore size of 200 meshes). The spleen was triturated with disposable syringe needle so that the cells can be filtered out from the metal filters. The cell suspension was centrifuged at 1000 r/min (centrifugal radius 10 cm) for 3–5 min, and the supernatant was removed. The cells were suspended in the RPMI 1640 medium containing 10% fetal calf serum. Trypan blue staining indicated that the viable cell count was over  95%. The spleen cells density was adjusted to 5 × 10^6^–10^7^/mL, and the cells were incubated at 37°C in 5% CO_2_ incubator.

### 2.5. DNA Damage Assay

Primary spleen lymphocyte cells were cultured for 24 h and centrifuged for 3–5 min (1000 r/min centrifugal radius 10 cm). The cells were resuspended in the RPMI 1640 medium with no fetal calf serum. Trypan blue staining shows that the viable cell count was over 95%, and cell density was adjusted to 5 × 10^6^/mL. One ml of spleen cell suspension was added into each of 12 sterilization centrifuge tubes, which were randomly divided into 4 groups of 3 tubes. Twenty-five *μ*L H_2_O_2_  solution of different concentrations (25, 50, and 125 *μ*mol/L) was added into tubes of the 3 H_2_O_2_ groups, and equal volume of PBS was added into the blank control group and all tubes were incubated at 4°C for 20 min. Then, single-cell gel electrophoresis (SCGE) was performed.

### 2.6. Assay for FR Pretreatment *In Vitro*

A density of 1 × 10^7^/mL of primary cells was prepared. One mL of lymphocyte suspension was added into each of the 18 centrifuged tubes, which were randomly divided into control group, H_2_O_2_ treatment group, and the FR treatment groups I, II, III, and IV, with 3 tubes in each group. The cells in the FR groups received the treatment of different concentrations of FR extracts (1 mL) and incubated at 37°C in 5% CO_2_ for 60 min, followed by the treatment of 25 *μ*L H_2_O_2_ solution (50 *μ*mol/L) at 4°C for 20 min, while the cells in the H_2_O_2_ treatment group only received the treatment of 25 *μ*L H_2_O_2_ solution. The control group cells received PBS treatment. After the treatment, the cells were harvested for SCGE.

### 2.7. Single-Cell Gel Electrophoresis

Improved methods were used in the study such as Singh, cell preparation in alkaline conditions, alkali treatment, electrophoresis, neutralization, EB staining, reviewing, and analyzing. A laser confocal microscope was used to analyze the morphology of the cells at a wavelength of 488 nm (10∗20 times magnification). One hundred randomly selected cells were used to calculate the DNA migration rate (tailing rate), and the total length (maximum length of the direction of comet) was considered as the tail length.

### 2.8. Animal Treatments

Different groups of animals were used to explore the effects of  FR on KBrO_3_-induced oxidative stress and 8-OHdG induction in the renal tissue of rats. Thirty male Wistar rats were randomly divided into 5 groups (6 rats in each group). Group I received saline injection intraperitoneally (0.85% NaCl) at a dose of 10 mL/kg body weight. Group II received a single intraperitoneal injection of KBrO_3_ at a dose of 125 mg/kg body weight. Group III received pretreatment with FR by gavage once a day for 5 days at a dose of 150 mg/kg body weight, and groups IV and V received the pretreatment with FR by gavage once a day for 5 days at a dose of 300 mg/kg body weight. After the last treatment with FR, the rats of groups II, III, and IV received a single intraperitoneal injection of KBrO_3_ at a dose level of 125 mg/kg body weight. Twenty-four hours later, the rats were sacrificed by cervical dislocation.

Blood samples were collected in heparinised tubes, and the plasma was separated by centrifugation at 2000 ×g for 10 min. The tissues (liver and kidney) were isolated and immediately transferred to ice-cold containers containing 0.9% sodium chloride for various analyses. A known amount of tissue was weighed and homogenized in appropriate buffer for the evaluation of various biochemical parameters.

### 2.9. Biochemical Assays

The antioxidant status was evaluated by measuring the levels of reduced glutathione (GSH) by Khan's method [14] with minor modifications. The activities of glutathione peroxidase (GPx) and SOD were measured as described by Rotruck et al. [15] and Chen et al. [16], respectively. The content of H_2_O_2_ was assayed by the H_2_O_2_-mediated horseradish peroxidase-dependent oxidation of phenol red by the method of Pick and Keisari [17]. The activity of catalase (CAT) was measured by the method as described by Sinha [18] with minor modifications. The levels of 8-OHdG in plasma and tissues were measured according to the instruction provided in the assay kit for 8-OHdG.

### 2.10. Statistical Analysis

Statistical analysis was performed using SPSS15.0. One-way ANOVA (*P* < 0.05) and *q* test (Student-Newman-Keuls) were used to compare the means among the groups with measurement data after homogeneity testing for homogeneity of variance, and we analyzed enumeration data with chi-square statistics. The level of significance was set at *P* < 0.05.

## 3. Results

The results of the effects of different concentrations of  H_2_O_2_ on DNA damage *in vitro* were summarized in Table 1. After treatment with 25 *μ*mol/L H_2_O_2_ for 20 min, the number of comet tail cells (88%) was significantly increased as compared with the result (15%) of the control group. When the concentration of H_2_O_2_ reached 50 and 125 *μ*mol/L, the numbers of comet cells were 100%. The comet tail length indicated the severity of DNA damage. The tail lengths of comet cells treated with H_2_O_2_ were significantly longer than those of the control cells (Table 1).

**Figure 1:**
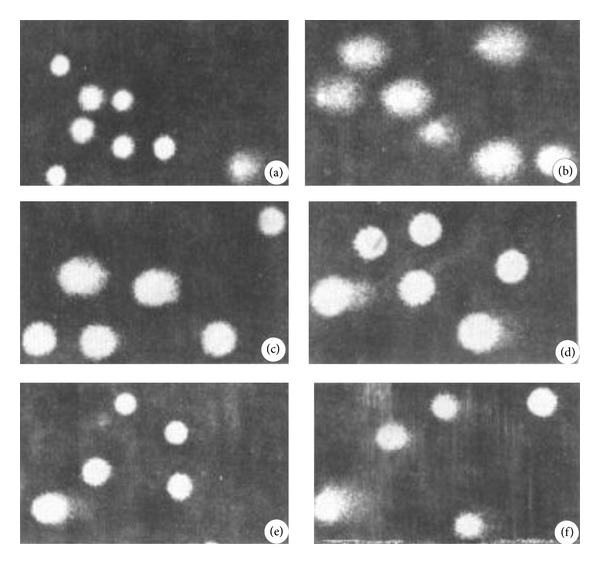
The effects of FR on lymphocyte DNA damage induced by H_2_O_2_  (*n* = 100). (a) Negative control; (b) positive control; (c) a dilution of 80 times of FR (1 g/mL); (d) a dilution of 40 times of FR (1 g/mL); (e) a dilution of 20 times of FR (1 g/mL); (f) a dilution of 10 times of FR (1 g/mL).

The effects of pretreatment of lymphocytes with FR were shown in Table 2. The data showed that FR of various concentrations could significantly reduce DNA damage induced by H_2_O_2_ in spleen lymphocytes. The protective effects of FR were significantly increased with increased FR concentrations from 20 to 80 dilution fold as compared with the positive control group (*P* < 0.01), and the maximum protective effect was observed when FR was diluted 20 times. However, when the concentration of FR was 10 dilution folds, the results of comet cell tailing rate and total comet length were significantly increased as compared with the positive control group (*P* < 0.01) (Figure 1, Table 2).

The effects of FR on KBrO_3_-mediated oxidative stress and antioxidant ability in rats were shown in Tables 3, 4, 5, 6, 7, and 8. The treatment of KBrO_3_ alone significantly reduced the activities of SOD, CAT, and GPx and the levels of GSH compared with those of the saline-treated control group, and the pretreatment of FR at 150 mg/kg body weight and 300 mg/kg body weight partially recovered the activities of SOD, CAT, and GPx and the levels of GSH in a concentration-dependent manner. However, treatment of FR alone did not produce any effects on the activities of the antioxidant enzymes.

The levels of hydroperoxides and 8-OHdG were significantly elevated in plasma and tissues of the rats treated with KBrO_3_ as compared with the control rats. Pretreatment of FR significantly decreased the levels of hydroperoxides as compared with KBrO_3_-treated rats. 

## 4. Discussion

This study demonstrated that FR protected against oxidative DNA damage and to varying degrees reversed the damages caused by oxidative stress via its antioxidant activities. These findings support the hypothesis that FR exerted a protective effect *in vivo* as well as *in vitro*.

We evaluated the protective effects of  FR against oxidative DNA damage by H_2_O_2_ with spleen lymphocytes being based on the following considerations. Firstly, oxidative DNA damage is closely related to aging, and immune senescence plays an important role in aging. During the process of aging, there will be varying degrees of degradation in the immune organs such as spleen and thymus, so it is of great significance to protect the immune cells to avoid oxidative DNA damage caused by chemicals [19]. Secondly, spleen is the largest immune organ in the body, and the cell model is easy to build. Thirdly, spleen lymphocyte models are widely used in experiment to study the protective effects of traditional Chinese medicines.

FR has been reported to contain flavonoid glycosides, phenols, amino acids, organic acids, and carbohydrates [3]. The major antioxidant active constituents of  FR are flavonoid glycosides and phenols, which display free radical-scavenging activity and antioxidant properties [20–22]. The observed chemopreventive activity of FR in this study suggested that the protective effects of FR may be attributed to the action of these compounds in FR [23, 24].

 In evaluating the effects of FR on lymphocyte DNA damage induced by H_2_O_2_, we have tried different dilutions of FR and found 10 times dilution working in a different way as compared with 20 times dilution. The difference between their values was statistically significant in terms of the number of comet cells but not statistically significant in terms of the distance of comet tail (Table 2). This is indeed a very interesting phenomenon and might provide evidence to the double-edged sword theory of antioxidants and reductive stress.

 Antioxidants may promote oxidation at high concentrations. Skibola CF and Smith MT have found the potentially toxic effects of excessive flavonoid intake. At high doses, flavonoids may act as mutagens, prooxidants that generate free radicals, and as inhibitors of key enzymes involved in hormone metabolism. Thus, at high doses, the adverse effects of flavonoids may outweigh their beneficial ones. The unborn fetus may be especially at risk, since flavonoids readily cross the placenta [25, 26]. 

 Redox imbalance in cells can lead either to oxidative or to reductive stress. Oxidative stress has been extensively studied for many years, and its possible clinical ramifications have been explored in considerable depth. Reductive stress, by contrast, has not been widely recognised. Yet reductive stress is probably both common and of clinical importance: indeed, reductive stress plus oxygen rather than oxidative stress may be the most common mechanism leading to the generation of reactive oxygen species (ROS). One possible link between the two may be the reduction of Fe^3+^ and its liberation from ferritin. The reduced metal could catalyse ROS generation [27].

Our results also showed decreased activities of enzymatic antioxidants SOD, CAT and GPx, and the levels of non-enzymatic antioxidant GSH in circulation, liver and kidney of KBrO_3_-treated rats. ROS generated in tissues are normally scavenged by enzymatic and non-enzymatic antioxidants [28, 29]. Antioxidant defense system protects the aerobic organism from the deleterious effects of reactive oxygen metabolites [30].

Glutathione is a crucial component of the antioxidant defense mechanism and it functions as a direct reactive free radical scavenger [31, 32]. The liver is the major organ with the highest content of GSH, which is transferred to kidney by distinct GSH transport system [33, 34]. The decreased levels of GSH in circulation and tissues in KBrO_3_-treated rats may due to enhanced utilization during detoxification of KBrO_3_. GPx and CAT, which act as preventive antioxidants and SOD, a chain-breaking antioxidant, play important roles in protection against the deleterious effects of lipid peroxidation [35]. Decreases in the activities of SOD, CAT and GPx in plasma, liver and kidney of KBrO_3_-treated rats may due to the decreased synthesis of enzymes or oxidative inactivation of the enzyme proteins. In the present study, increased lipid peroxidation associated with decreased antioxidant status in KBrO_3_-treated rats could therefore give rise to insufficient antioxidant potential.

The antioxidant effects of FR may be due to different causes: firstly, flavonoid's high diffusion into the membranes [36] allowed it to scavenge oxyradicals at several sites throughout the lipid bilayer; secondly, its pentahydroxyflavone structure allowed it to chelate metal ions via the orthodihydroxy phenolic structure, thereby scavenging lipid alkoxyl and peroxyl radicals [37, 38]. In spite of the free radical scavenging activities, flavanoid glycosides and phenols in FR might be also involved in the indirect induction of detoxifying genes [39], which might promote detoxification of KBrO_3_ and decrease their toxicity. *In vivo* studies have shown that flavanoid glycosides inhibited Fe^2+^-induced lipid peroxidation in the rat liver [40]. It is suggested that the lipid peroxidative indices were probably attenuated by the chain-breaking action of flavanoid in the free radical process of the oxidation of membrane lipids.

Our results also showed that the kidney was the major organ with the higher content of 8-OHdG after KBrO_3_ treatment, suggesting that the kidney is the main target organ of KBrO_3_-induced DNA oxidation. DNA damage was significantly decreased after the treatment of FR, which may be attributed to the antioxidant property of phenols in FR, as phenols are known to bind DNA at sites that would normally react with the active metabolites of carcinogen during carcinogen-DNA binding, a crucial step for initiation of carcinogenesis [41–43]. Alternatively, when the phenols bind to DNA, their molecules might be positioned in such a way so as to effectively scavenge reactive intermediates that approach the critical sites on DNA, or phenols may directly interact with the ultimate reactive metabolites of carcinogen by donating their electrons and rendering it inactive [44]. Dok-Go et al. demonstrated phenolsact in many cell-free experimental systems to scavenge reactive oxygen radicals and reduce oxidative DNA damage [45].

In summary, our data demonstrated that FR protected against KBrO_3_ toxicity by decreasing oxidant status and DNA damage and increasing the antioxidant status, indicating that FR possesses a spectrum of antioxidant and DNA-protective properties. However, further investigations are necessary to elucidate the precise mechanisms of protection of FR against KBrO_3_ toxicity, and the potential effects of FR against other carcinogens should be explored prior to evaluating as a chemopreventive agent against carcinogenesis.

## Figures and Tables

**Table 1:** DNA damage of lymphocytes induced by H_2_O_2_ (*n* = 100).

Treatment groups	Number of comet cells (%)	Distance of comet tail (*μ*m) $\overset{-}{x}\pm S$
H_2_O_2_ 25 *μ*mol/L	88.0**	42.89 ± 9.25**
H_2_O_2_ 50 *μ*mol/L	100.0**	50.45 ± 8.64**
H_2_O_2_ 125 *μ*mol/L	100.0**	53.28 ± 9.58**

Control	15.0	28.12 ± 6.75
	*χ* ^2^ = 298.5	*F* = 259.2

**P < 0.01 compared with the control.

**Table 2:** The effects of FR on lymphocyte DNA damage induced by H_2_O_2_ (*n* = 100).

Treatment groups	Number of comet cells (%)	Distance of comet tail (*μ*m) (mean ± SD)
Negative (A)	16.0	21.35 ± 2.54
Positive (B)	98.0	49.23 ± 7.27
Dilution ratio of FR (1 g/mL)		
80 times (C)	82.0*	40.51 ± 8.33*
40 times (D)	62.0**	35.29 ± 7.81**
20 times (E)	28.0**	25.45 ± 4.65**
10 times (F)	45.0^∗∗#^	34.36 ± 8.12**

	*χ* ^2^ = 213.7	*F* = 163.5

*P < 0.05, **P < 0.01, compared with the positive control. ^#^ *P* < 0.05, compared with E group.

**Table 3:** The levels of hydroperoxides in plasma and tissues.

Treatment groups	Plasma (×10^−5^ mM)	Kidney (mM/100 g tissue)	Liver (mM/100 g tissue)
Saline (control)	10.26 ± 0.71	38.59 ± 2.43	43.27 ± 2.83
KBrO_3_ (125 mg/kg body weight)	20.38 ± 1.29^△△^	73.50 ± 5.28^△△^	63.26 ± 4.29^△△^
FR (150 mg/kg body weight) + KBrO_3_	12.86 ± 0.83**	59.32 ± 4.22*	49.55 ± 3.36*
FR (300 mg/kg body weight) + KBrO_3_	11.60 ± 0.78**	53.78 ± 3.73**	46.27 ± 3.69*
FR (300 mg/kg body weight) alone	10.52 ± 0.73	36.92 ± 2.79	42.65 ± 3.30

Values were expressed as mean ± SD, *n* = 6. The dose of KBrO_3_ was 125 mg/kg body weight in each group.

^△^ *P* < 0.05 and ^△△^ *P* < 0.01 compared with the control.

*P < 0.05 and **P < 0.01 compared with KBrO_3_-treated rats.

**Table 4:** The activities of SOD in hemolysate and tissues.

Treatment groups	Hemolysate U^A^	Kidney U^B^	Liver U^B^
Saline (control)	4.82 ± 0.35	8.64 ± 0.65	9.28 ± 0.62
KBrO_3_ (125 mg/kg body weight)	2.65 ± 0.17^△△^	5.14 ± 0.35^△△^	6.50 ± 0.45^△△^
FR (150 mg/kg body weight) + KBrO_3_	3.48 ± 0.24*	6.82 ± 0.44*	7.22 ± 0.52*
FR (300 mg/kg body weight) + KBrO_3_	3.85 ± 0.27**	7.37 ± 0.48**	8.35 ± 0.56**
FR (300 mg/kg body weight) alone	4.26 ± 0.32	8.43 ± 0.57	8.95 ± 0.59

Values were expressed as mean ± SD, *n* = 6. The dose of KBrO_3_ was 125 mg/kg body weight in each group.

U^A^: enzymes required for 50% inhibition of NBT reduction min/mg Hb; U^B^: enzymes required for 50% inhibition of NBT reduction min/mg protein.

^△^ *P* < 0.05 and ^△△^ *P* < 0.01 compared with the control group.

*P < 0.05 and **P < 0.01 compared with KBrO_3_-treated rats.

**Table 5:** The activities of CAT in hemolysate and tissues.

Treatment groups	Hemolysate U^A^	Kidney U^B^	Liver U^B^
Saline (control)	3.65 ± 0.27	22.71 ± 1.25	68.26 ± 3.15
KBrO_3_ (125 mg/kg body weight)	1.59 ± 0.13^△△^	13.23 ± 0.79^△△^	49.75 ± 2.62
FR (150 mg/kg body weight) + KBrO_3_	2.86 ± 0.20*	14.82 ± 0.82	60.35 ± 2.79*
FR (300 mg/kg body weight) + KBrO_3_	3.19 ± 0.25**	15.37 ± 0.85	62.85 ± 2.80*
FR (300 mg/kg body weight) alone	3.48 ± 0.31	21.28 ± 1.24	66.27 ± 3.02

Values were expressed as mean ± SD, *n* = 6. The dose of KBrO_3_ was 125 mg/kg body weight in each group.

U^A^: *µ* moles of H_2_O_2_ utilized/min/mg Hb; U^B^: *µ* moles of H_2_O_2_ utilized/min/mg protein.

^△^ *P* < 0.05 and ^△△^ *P* < 0.01 compared with the control group.

*P < 0.05 and **P < 0.01 compared with KBrO_3_-treated rats.

**Table 6:** The activities of GPx in hemolysate and tissues.

Treatment groups	Hemolysate U^A^	Kidney U^B^	Liver U^B^
Saline (control)	26.28 ± 1.52	10.06 ± 0.61	11.12 ± 0.74
KBrO_3_ (125 mg/kg body weight)	13.75 ± 0.94^△△^	6.15 ± 0.39^△△^	7.68 ± 0.46^△△^
FR (150 mg/kg body weight) + KBrO_3_	17.29 ± 1.12*	7.67 ± 0.45	9.10 ± 0.65*
FR (300 mg/kg body weight) + KBrO_3_	23.82 ± 1.50**	8.74 ± 0.56*	10.06 ± 0.63*
FR (300 mg/kg body weight) alone	26.65 ± 1.63	9.82 ± 0.59	10.55 ± 0.70

Values were expressed as mean ± SD, *n* = 6. The dose of KBrO_3_ was 125 mg/kg body weight in each group.

U^A^: *µ* moles of GSH utilized/min/mg Hb; U^B^: *µ* moles of GSH utilized/min/mg protein.

^△^ *P* < 0.05 and ^△△^ *P* < 0.01 compared with the control group.

*P < 0.05 and **P < 0.01 compared with KBrO_3_-treated rats.

**Table 7:** The levels of GSH in plasma and tissues.

Treatment groups	Plasma (mg/dL)	Kidney (mg/100 g tissue)	Liver (mg/100 g tissue)
Saline (control)	35.28 ± 2.57	92.28 ± 5.74	125.72 ± 8.29
KBrO_3_ (125 mg/kg body weight)	20.85 ± 1.62^△△^	58.20 ± 4.12^△△^	78.27 ± 5.71^△△^
FR (150 mg/kg body weight) + KBrO_3_	28.24 ± 1.72**	71.39 ± 4.81*	107.22 ± 6.26**
FR (300 mg/kg body weight) + KBrO_3_	31.39 ± 1.83**	79.55 ± 5.88**	118.82 ± 7.20**
FR (300 mg/kg body weight) alone	34.20 ± 1.96	89.26 ± 6.26	126.18 ± 8.75

Values were expressed as mean ± SD, *n* = 6. The dose of KBrO_3_ was 125 mg/kg body weight in each group.

^△^ *P* < 0.05 and ^△△^ *P* < 0.01 compared with the control group.

*P < 0.05 and **P < 0.01 compared with KBrO_3_-treated rats.

**Table 8:** The levels of 8-OHdG in plasma and tissues.

Treatment groups	Plasma (ng/mL)	Kidney (ng/mL)	Liver (ng/mL)
Saline (control)	0.69 ± 0.08	1.12 ± 0.15	0.92 ± 0.12
KBrO_3_ (125 mg/kg body weight)	9.65 ± 1.26^△△^	21.65 ± 2.94^△△^	13.73 ± 2.13^△△^
FR (150 mg/kg body weight) + KBrO_3_	5.27 ± 0.68**	12.45 ± 1.57*	7.82 ± 1.28**
FR (300 mg/kg body weight) + KBrO_3_	2.59 ± 0.23**	3.38 ± 0.45**	2.73 ± 0.37**
FR (300 mg/kg body weight) alone	0.75 ± 0.10	1.05 ± 0.18	0.89 ± 0.15

Values were expressed as mean ± SD, *n* = 6. The dose of KBrO_3_ was 125 mg/kg body weight in each group.

^△^ *P* < 0.05 and ^△△^ *P* < 0.01 compared with the control group.

*P < 0.05 and **P < 0.01 compared with KBrO_3_-treated rats.

## Abbreviations

FR: *Fructus rhodomyrti*
SCGE: Single-cell gel electrophoresis
ROS: Reactive oxygen species
SOD: Superoxide dismutase
CAT: Catalase
GSH: Reduced glutathione
GPx: Glutathione peroxidase
8-OHdG: 8-Hydroxy-2′-deoxyguanosine.
